# Glucocorticoid Receptor Antagonism Improves Glucose Metabolism in a Mouse Model of Polycystic Ovary Syndrome

**DOI:** 10.1210/jendso/bvad162

**Published:** 2023-12-20

**Authors:** Sheng Li, Zhixiong Ying, Max Gentenaar, Patrick C N Rensen, Sander Kooijman, Jenny A Visser, Onno C Meijer, Jan Kroon

**Affiliations:** Department of Medicine, Division of Endocrinology, Leiden University Medical Center, 2333ZA Leiden, the Netherlands; Einthoven Laboratory for Experimental Vascular Medicine, Leiden University Medical Center, 2333ZA Leiden, the Netherlands; Department of Medicine, Division of Endocrinology, Leiden University Medical Center, 2333ZA Leiden, the Netherlands; Einthoven Laboratory for Experimental Vascular Medicine, Leiden University Medical Center, 2333ZA Leiden, the Netherlands; Department of Medicine, Division of Endocrinology, Leiden University Medical Center, 2333ZA Leiden, the Netherlands; Einthoven Laboratory for Experimental Vascular Medicine, Leiden University Medical Center, 2333ZA Leiden, the Netherlands; Department of Medicine, Division of Endocrinology, Leiden University Medical Center, 2333ZA Leiden, the Netherlands; Einthoven Laboratory for Experimental Vascular Medicine, Leiden University Medical Center, 2333ZA Leiden, the Netherlands; Department of Medicine, Division of Endocrinology, Leiden University Medical Center, 2333ZA Leiden, the Netherlands; Einthoven Laboratory for Experimental Vascular Medicine, Leiden University Medical Center, 2333ZA Leiden, the Netherlands; Department of Internal Medicine, Erasmus MC, University Medical Center Rotterdam, 3015 GD Rotterdam, the Netherlands; Department of Medicine, Division of Endocrinology, Leiden University Medical Center, 2333ZA Leiden, the Netherlands; Einthoven Laboratory for Experimental Vascular Medicine, Leiden University Medical Center, 2333ZA Leiden, the Netherlands; Department of Medicine, Division of Endocrinology, Leiden University Medical Center, 2333ZA Leiden, the Netherlands; Einthoven Laboratory for Experimental Vascular Medicine, Leiden University Medical Center, 2333ZA Leiden, the Netherlands

**Keywords:** androgen receptor, dihydrotestosterone, glucocorticoid receptor, metabolism, polycystic ovary syndrome

## Abstract

**Context:**

Polycystic ovary syndrome (PCOS) is a complex metabolic disorder associated with obesity, insulin resistance, and dyslipidemia. Hyperandrogenism is a major characteristic of PCOS. Increased androgen exposure is believed to deregulate metabolic processes in various tissues as part of the PCOS pathogenesis, predominantly through the androgen receptor (AR). Notably, various metabolic features in PCOS are similar to those observed after excess glucocorticoid exposure.

**Objective:**

We hypothesized that glucocorticoid receptor (GR) signaling is involved in the metabolic symptoms of PCOS.

**Methods:**

In a PCOS model of chronic dihydrotestosterone (DHT) exposure in female mice, we investigated whether GR signaling machinery was (de)regulated, and if treatment with a selective GR antagonist alleviated the metabolic symptoms.

**Results:**

We observed an upregulation of GR messenger RNA expression in the liver after DHT exposure. In white adipose tissues and liver we found that DHT upregulated *Hsd11b1*, which encodes for the enzyme that converts inactive into active glucocorticoids. We found that preventive but not therapeutic administration of a GR antagonist alleviated DHT-induced hyperglycemia and restored glucose tolerance. We did not observe strong effects of GR antagonism in DHT-exposed mice on other features like total fat mass and lipid accumulation in various tissues.

**Conclusion:**

We conclude that GR activation may play a role in glucose metabolism in DHT-exposed mice.

Polycystic ovary syndrome (PCOS) is a common hormonal disorder in women leading to infertility and is estimated to have a global prevalence of 6% to 20% [1]. In PCOS, long-term and continuous exposure to elevated levels of androgens are considered the major driving force of the clinical features [2]. In addition to infertility, PCOS is associated with metabolic symptoms such as obesity, insulin resistance, and dyslipidemia [1, 3, 4]. Insulin resistance can also induce elevated androgen levels by reducing sex hormone binding globulin and thereby resulting in increased free androgen levels and increased androgen signaling [5, 6]. At the molecular level, androgens primarily exert their effects through the androgen receptor (AR), and preclinical studies in AR knockout mice have shown that AR signaling is crucial in the development of PCOS-related symptoms [7, 8]. The AR undergoes a conformational change on ligand binding and translocates to the nucleus, where it exerts its transcriptional effects [9, 10]. Besides the involvement of androgen signaling, the underlying mechanisms of how metabolic symptoms in PCOS develop still remain largely unknown. We previously found in male mice that androgen signaling can strongly influence the outcome of glucocorticoid signaling [11]. Glucocorticoid receptor (GR) signaling is known to play a major role in various metabolic process [12, 13], including lipid metabolism [14] and glucose metabolism [15-17]. Notably, many of the clinical features of PCOS overlap with those of excess glucocorticoid exposure [18].

Glucocorticoid signaling is regulated at several levels. At an enzymatic level active glucocorticoid levels are controlled by 11 beta-hydroxysteroid dehydrogenase type 1 (11β-HSD1) [19], an enzyme that converts inactive glucocorticoids into active glucocorticoids, and that is known to play a role in the development of obesity [20]. It has been shown that the androgen dihydrotestosterone (DHT) increases the expression of 11β-HSD1 in mouse and human adipose tissue, thereby influencing local turnover of corticosterone/cortisol [21, 22]. The enzyme 11 beta-hydroxysteroid dehydrogenase type 2 inactivates glucocorticoids, and its expression is more restricted [18]. In addition to enzymatic regulation, the outcome of GR signaling is dependent on interaction with coregulatory proteins such as nuclear receptor coactivator 1 (NCOA1/SRC1) and nuclear receptor coactivator 2 (NCOA2/SRC2) [23, 24]. NCOA1 and NCOA2 were shown to play an important role in metabolic homeostasis [25-28]. It is important to note that many of these coregulatory proteins are also involved in AR signaling [29]. Given the similarities in metabolic symptoms in PCOS and upon excess glucocorticoid exposure, we hypothesized that glucocorticoid signaling may contribute to the metabolic symptoms observed in PCOS [30, 31]. In this study, we evaluated if GR machinery is altered in female mice on DHT exposure, and explored whether GR antagonism can be used to alleviate DHT-induced metabolic symptoms. For this, we made use of the recently developed GR antagonist CORT125134 (relacorilant), that—in contrast to classic GR antagonist RU486—lacks cross-reactivity with the AR and the progesterone receptor (PR) [32, 33].

## Materials and Methods

### Cell Culture and Reporter Assay

Human HEK293T cells were seeded at 80 000 cells per well in 24-well plates in Dulbecco’s modified Eagle’s medium + GlutaMAX with 10% charcoal-stripped fetal bovine serum supplemented with penicillin/streptomycin. The next day cells were transfected using one of the following mixtures: I) 25 µL OPTIMEM, 10 ng human GR, 25 ng TAT1-luciferase, 1 ng CMV-renilla, 265 ng pcDNA, and 1.25 µL Fugene HD transfection reagent (Promega); II) 25 µL OPTIMEM, 10 ng human PR, 25 ng TAT3-luciferase, 1 ng CMV-renilla, 265 ng pcDNA, and 1.25 µL Fugene HD transfection reagent; and III) 25 µL OPTIMEM, 10 ng human AR, 25 ng TAT1-luciferase, 1 ng CMV-renilla, 265 ng pcDNA, and 1.25 µL Fugene HD transfection reagent. Cells were pretreated for 1 hour with 0.1 to 1000 nM CORT125134 (relacorilant) (for GR signaling assays) or 10 to 1000 nM CORT125134 (for PR and AR signaling assays), and were subsequently treated with agonists for the GR (3 nM dexamethasone), PR (10 nM progesterone), and AR (100 nM DHT). After 24 hours, cells were harvested and firefly- and renilla-luciferase signals were measured using a dual-luciferase assay (Promega). Data are normalized to agonist treatment and half maximal inhibitory concentration values were calculated using nonlinear regression. All conditions were performed in technical triplicate.

### Animals

This animal study was approved by the ethics committee of Leiden University Medical Center. Female C57BL6/J mice were purchased from Charles Rivers Laboratories and group-housed in conventional cages with a 12-hour:12-hour light/dark environment and had ad libitum access to water and a synthetic low-fat diet for 90 days.

### Animal Experiment

We used the androgen DHT to induce PCOS-like characteristics in female mice [34]. Female mice aged between 4 and 5 weeks were implanted subcutaneously under isoflurane anesthesia with either a blank 1-cm Silastic tube (inner diameter, 1.58 mm; outer diameter, 2.41 mm) or with a tube containing 10 mg DHT. Silastic implants were made in house and are known to provide a steady-state steroid hormone release for a period up to 6 months [35]. As a quality control, the presence of DHT powder was confirmed post euthanasia (12 weeks after implantation of silastic tubes).

We compared female mice with blank vs DHT implants to investigate the expression of GR-related factors in diverse metabolic tissues. We examined the role of GR signaling in the development of DHT-induced symptoms by feeding mice either a low-fat diet or low-fat diet supplemented with the selective GR antagonist CORT125134 (relacorilant) for a period of 90 days (500 mg per kg diet, resulting in an estimated dose of 60 mg/kg/day; “preventive” group). In parallel, we investigated GR antagonism in mice with an established PCOS-like metabolic phenotype as a result of DHT exposure, by administering 60 mg/kg/day CORT125134 or solvent (10% dimethyl sulfoxide, 0.5% Tween-80, 0.5% hydroxypropyl-methylcellulose in phosphate-buffered saline [PBS]) via daily oral gavage during weeks 9 to 12 (for a total of 21 days; “therapeutic” group). Overall, we evaluated the following groups: 1) control (N = 5), 2) control + preventive GR antagonism, N = 6), 3) control + therapeutic GR antagonism, 4) DHT (N = 6), 5) DHT + preventive GR antagonism (N = 6), and 6) DHT + therapeutic GR antagonism (N = 6).

### Body Mass and Body Composition Measurement

Body mass and total lean and fat mass were determined weekly by using an EchoMRI-100 analyzer.

### Plasma Biochemistry Measurements

At the end of week 12, blood plasma was collected from the tail vein from 6-hour fasted mice and these samples were used to measure plasma levels of insulin (Crystal Chem), glucose, triglycerides, and cholesterol (enzymatic kits from Roche Diagnostics).

### Oral Glucose Tolerance Test

In week 11, mice were fasted for 6 hours before a baseline blood glucose measurement was performed (t = 0). After this, 2 g/kg glucose was administered via oral gavage and blood glucose concentration was then measured at t = 15, 30, 60, 90, 120 minutes using an Accu-Check glucometer (Roche).

### Organ Uptake of Radiolabeled Triglyceride-derived Fatty Acids and Deoxyglucose

Triglyceride-rich lipoprotein–like emulsion particles (average size 80 nm) radiolabeled with glycerol tri[^3^H]oleate were prepared as previously described [36, 37]. Mice were fasted for 4 hours and injected intravenously in the tail vein with particles containing 1.0 mg triglyceride in combination with [^14^C]deoxyglucose in 200 µL PBS. After 15 minutes, mice were killed by CO_2_ inhalation and perfused with ice-cold PBS for 5 minutes before tissues were isolated to determine the ^3^H and ^14^C activity in various tissues. Tissue pieces were dissolved in 500 µL of Solvable (Perkin Elmer) overnight at 56 °C, and the ^3^H and ^14^C activity was determined using scintillation counting solution (Ultima Gold XR, Perkin Elmer).

### Histology

Ovaries, interscapular brown adipose tissue (iBAT) and gonadal white adipose tissue (gWAT) were fixed in 4% paraformaldehyde for 24 hours and stored in 70% ethanol before processing. Tissues were dehydrated, embedded in paraffin, cut into 5-µm sections and then stained with hematoxylin and eosin as previously described [38]. Lipid content in iBAT and average adipocyte cell size in gWAT were quantified using Image J software (version 1.48). In the ovaries, total number of corpora lutea (identified with consistent luteinized follicles and visible in serial sections) were quantified using a Zeiss Axio Observer A1 microscope. Large antral follicles were identified with a single large antrum. Follicles were assessed only in the sections where the oocyte's nucleolus were visible. Large antral follicles were categorized as unhealthy if they included a degenerate oocyte, and/or more than 5% of the granulosa cells were pyknotic in appearance, the percentage of unhealthy follicles per ovary was calculated. All large antral follicles were assessed for granulosa layer thickness and theca layer thickness using ImageJ software (version 1.48), as previously described [34]. One ovary could obtain more than one antral follicle. Several samples were lost during tissue processing, yielding ovaries of only N = 3 per group for analysis, and as we were thus underpowered we decided to not perform statistical analysis.

### Gene Expression Analysis

Total RNA was extracted from snap-frozen tissues using Tripure RNA isolation reagent (Roche). Complementary DNA was generated using M-MLV reverse-transcriptase (Promega). Quantitative reverse transcription–polymerase chain reaction was performed on a CFX96 PCR machine (Bio-Rad), and expression levels were normalized to the housekeeping gene *GAPDH*. Primer sequences: *Gapdh* Fwd: GGGGCTGGCATTGCTCTCAA; Rev: TTGCTCAGTGTCCTTGCTGGGG; *Gr* Fwd: CCCTCCCATCTAACCATCCT; Rev: ACATAAGCGCCACCTTTCTG; *Ar* Fwd: GCCTCCGAACTGTGGTATCC; Rev: CCTGGTACTGTCCAAACGCA; *Ncoa1* Fwd: GCGAGTCAAAGGGTGCAGTT; Rev: CCAGCCCGAAGCACATACA; *Ncoa2* Fwd: CGTCACCAACTGAGAAGCCA; Rev: GGACGGGTCAGAGGTGTTGTTTT; *Hsd11b1*: Fwd: AGTACACCTCGCTTTTGCGT; Rev: CTCTCTGTGTCCTTGGCCTC. *Hsd11b2*: Fwd: CACTCGAGGGGACGTATTGT; Rev: CGTTTCTCCCAGAGGTTCAC. Baseline expression (CT-values) for each gene is shown in Supplementary Table S1 [39].

### Statistical Analysis

Statistical analyses were performed with SPSS (version 25) and GraphPad Prism version 8.0.2. The following statistical analyses were used: 2-way analysis of variance with least significant difference post hoc test, unpaired *t* test, and linear mixed models. Data with 2 factors and multiple time points were analyzed using linear mixed models analysis that included independent variables as fixed factors. All data are presented as means ± SEM. *P* values of main effects and interactions of the analysis of variance are depicted in Supplementary Table S2 [39].

## Results

### Dihydrotestosterone Treatment Increased the Expression of Glucocorticoid Receptor Signaling Factors

**Figure 1.**
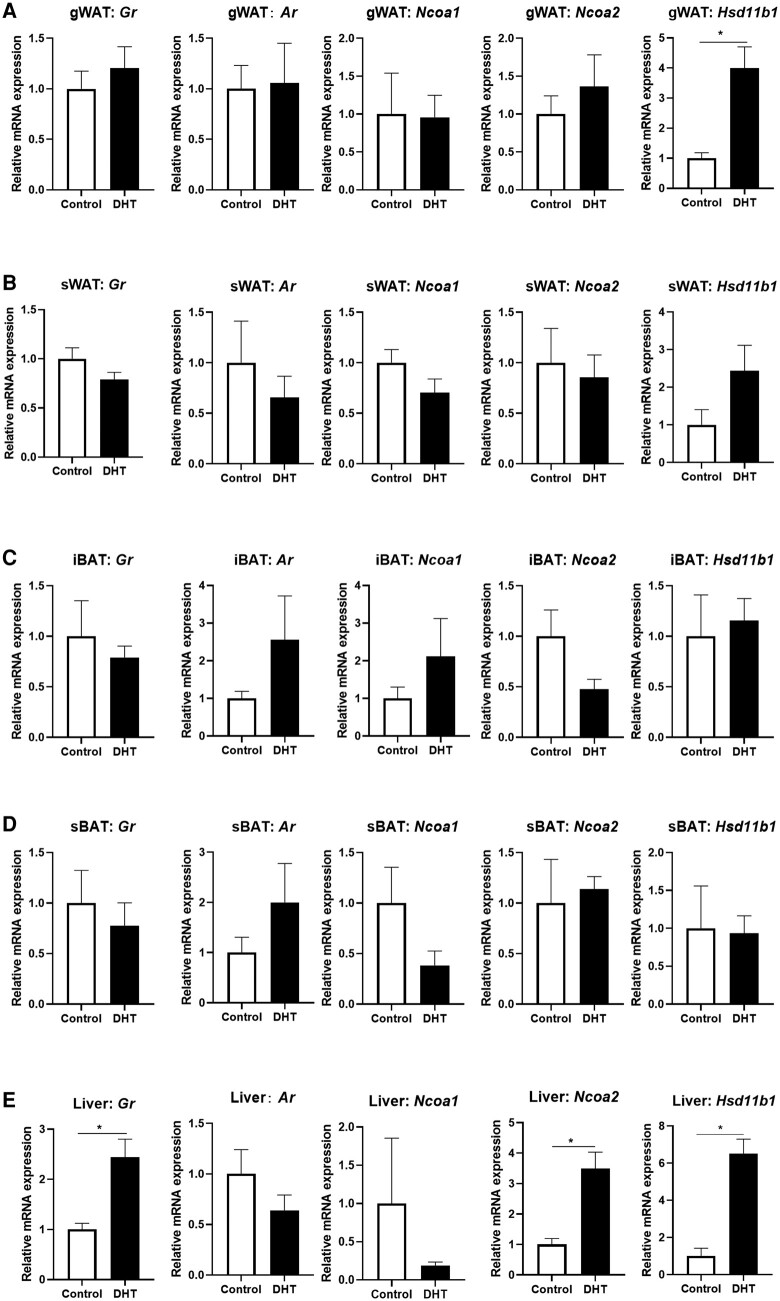
Effect of dihydrotestosterone (DHT) treatment on the expression of the glucocorticoid receptor (GR) and GR-related signaling factors. The messenger RNA (mRNA) expression of *Gr*, *Ar*, *Ncoa1*, *Ncoa2*, and *Hsd11b1* in A, gWAT; B, sWAT; C, iBAT; D, sBAT; and E, liver. Data are shown as mean ± SEM. N = 5 for the control group and N = 6 for the DHT group. Statistical significance is calculated using unpaired *t* test. *P less than .05 vs control.

We first set up to investigate the effect of DHT treatment on the expression of factors related to GR signaling in a range of tissues including gWAT, subcutaneous white adipose tissue (sWAT), iBAT, subscapular brown adipose tissue (sBAT), and liver. GR messenger RNA (mRNA) expression was elevated in the liver on DHT exposure, but was not significantly changed in other tissues (Fig. 1A-1E, first column). *Ar* expression was not changed after DHT exposure in any tissue (see Fig. 1A-1E, second column). Analysis of GR coactivators showed that *Ncoa1* expression in sBAT and liver was lower on DHT treatment, while *Ncoa2* expression was significantly increased in liver on DHT treatment (see Fig. 1A-1E, third and fourth column). Tissue-specific 11β-HSD1 activity determines the local active glucocorticoid level, and we found that DHT exposure significantly upregulated *Hsd11b1* mRNA in gWAT and liver, while a similar pattern was observed in sWAT (see Fig. 1A-1E, fifth column). *Hsd11b2*, the gene encoding for the enzyme 11β-HSD2 that inactivates glucocorticoids, was not expressed in any of these tissues, in neither vehicle condition, or on DHT exposure (see Supplementary Table S1) [39].

### Glucocorticoid Receptor Antagonism Does Not Influence Dihydrotestosterone-induced Features in the Ovary

**Figure 2.**
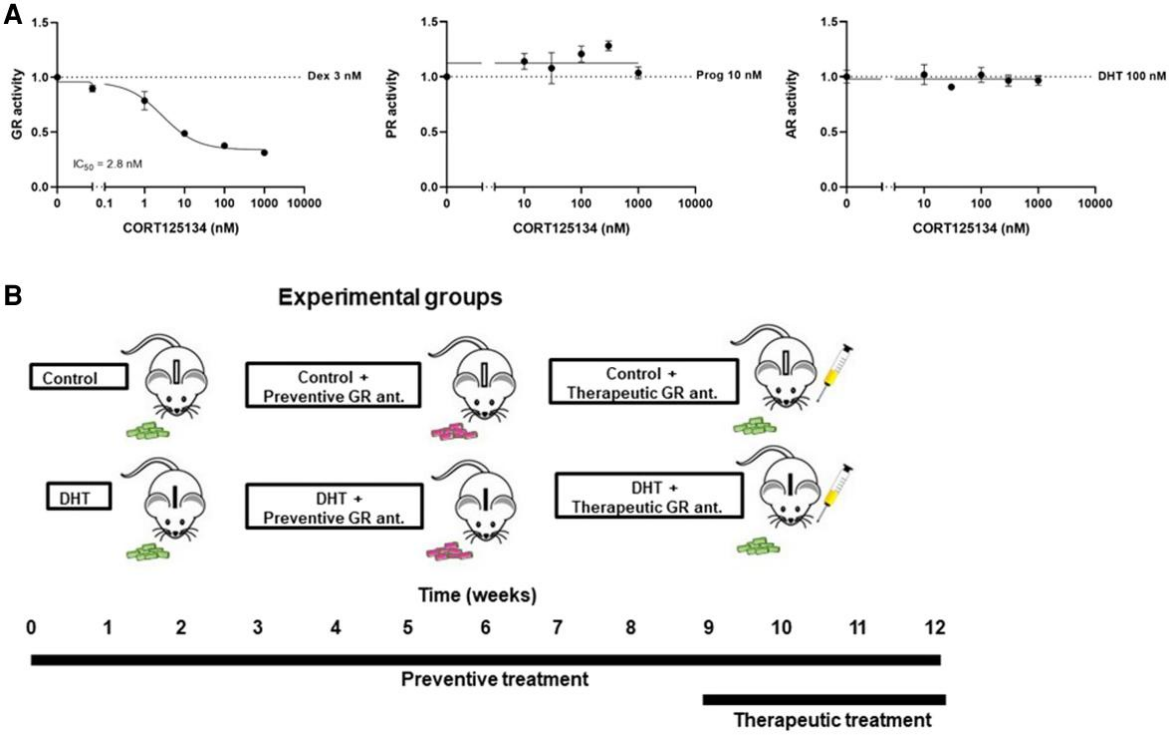
Experimental design to determine the effects of selective glucocorticoid receptor (GR) antagonism in a mouse model of elevated dihydrotestosterone (DHT) exposure. A, The effect of GR antagonist CORT125134 on GR, progesterone receptor (PR), and androgen receptor (AR) signaling in human HEK293T cells. B, Female mice were exposed to control or DHT-silica implants for 12 weeks. Mice were treated with a GR antagonist for 12 weeks via diet supplementation (“preventive treatment”) and during the last 3 weeks via oral gavage administration (“therapeutic treatment”). Body weight and composition were determined weekly; an oral glucose tolerance test was performed at week 11, and blood and tissues were collected after a 6-hour fast at the end of week 12.

**Figure 3.**
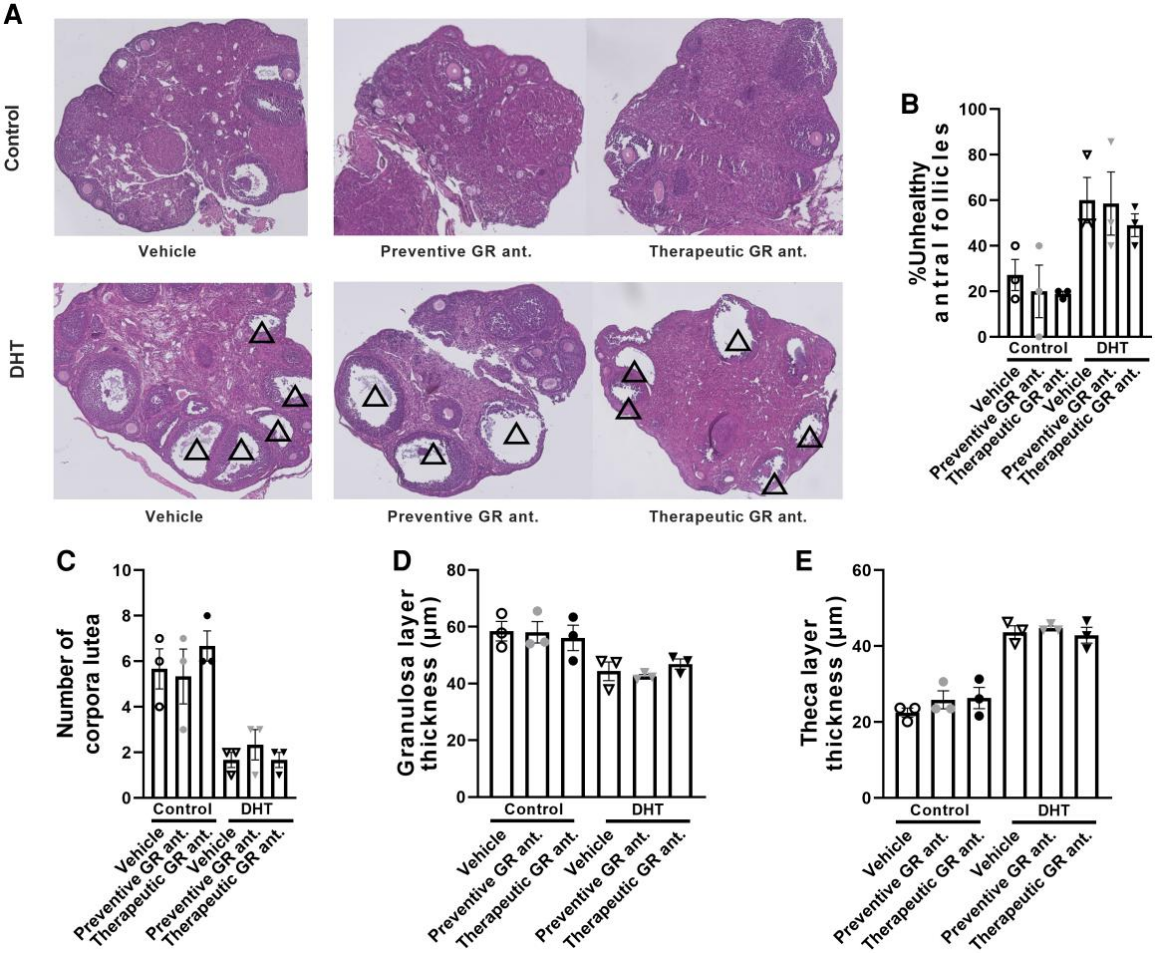
Dihydrotestosterone (DHT) exposure induces polycystic ovary syndrome (PCOS)-associated features in the mouse ovary. A, Histological sections of the ovary of control mice and DHT-exposed mice on preventive or therapeutic treatment with a glucocorticoid receptor (GR) antagonist. The PCOS-related features in the ovary are defined by the presence of multiple arrested large antral follicles (indicated with triangles). B, Proportion of unhealthy large antral follicles per ovary, and C, the number of corpora lutea. N = 3 per group. D, Average thickness of granulosa cell layer and E, theca layer, confirming PCOS-related features. Multiple follicles were averaged per mouse, as one ovary could contain multiple follicles. N = 3 mice per group. Data are shown as mean ± SEM.

We first confirmed the specificity of our GR antagonist CORT125134. Pretreatment with CORT125134 did not influence progesterone-induced PR signaling and DHT-induced AR signaling in human HEK293T cells, while GR signaling was potently antagonized with a half maximal inhibitory concentration of 2.8 nM (Fig. 2A). To investigate if GR antagonism influences the DHT-induced symptoms, we administered the GR-specific antagonist both to control and DHT-treated mice in a preventive and therapeutic treatment regimen (Fig. 2B). We first confirmed the PCOS-like features by histological analysis of the ovary. As expected, DHT treatment induced an increase in percentage of unhealthy antral follicles, a decrease in the number of corpora lutea, and a decrease in granulosa layer thickness and an increase in theca layer thickness, as compared to control mice (Fig. 3A-3E). GR antagonism, either in a preventive or therapeutic setting, did not seem to alter any of these DHT-induced features in the ovary (see Fig. 3A-3E).

### Preventive Glucocorticoid Receptor Antagonism Reduced Body Weight and Lean Mass Both in Control and Dihydrotestosterone-treated Mice

**Figure 4.**
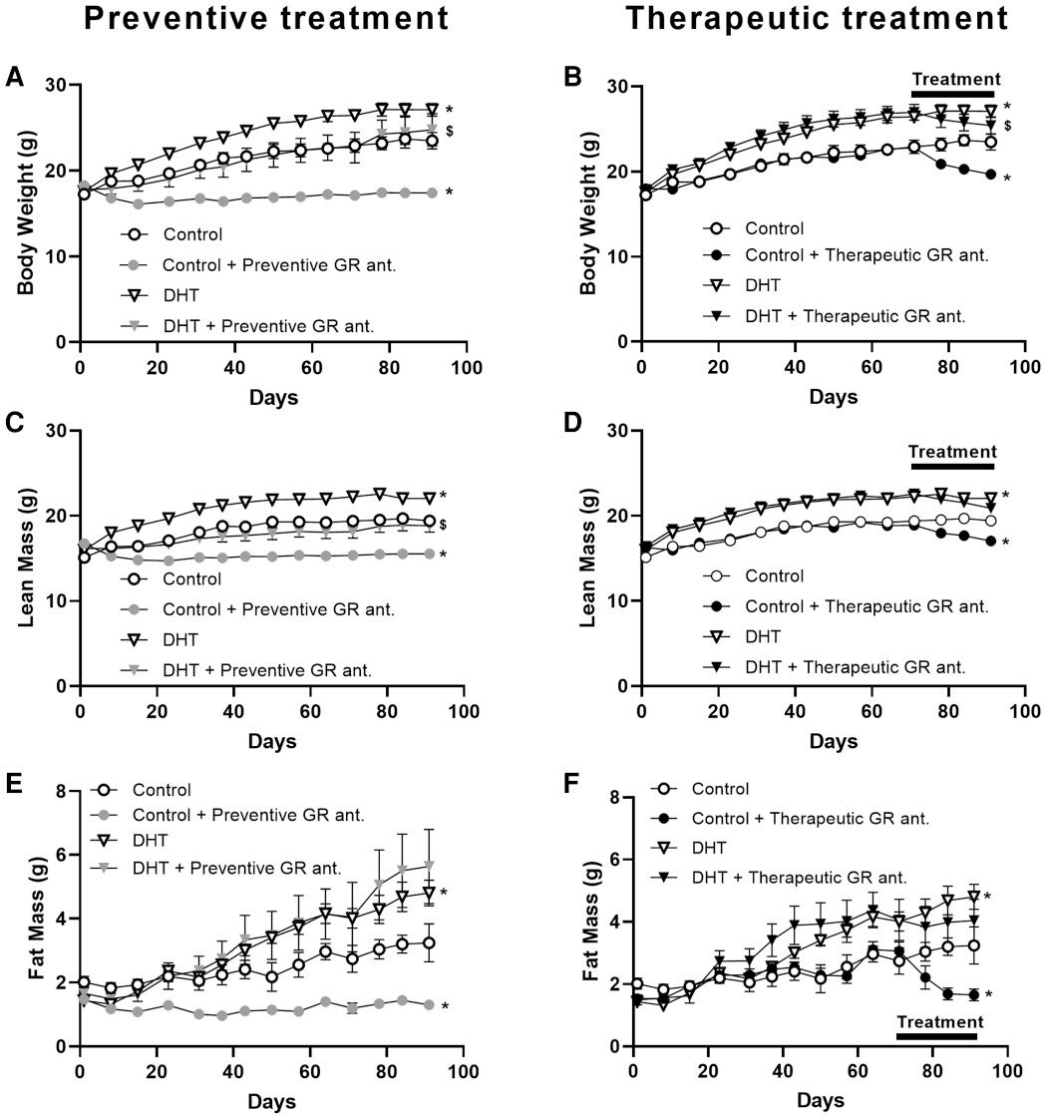
The effect of preventive and therapeutic glucocorticoid receptor (GR) antagonism on body weight, lean mass, and fat mass of control and dihydrotestosterone (DHT)-exposed mice. A and B, Body weight; C and D, lean mass; and E and F, fat mass. Data are shown as mean ± SEM. N = 5/6 per group. The control and DHT groups were plotted both in the preventive and the therapeutic graphs for clarity. Statistical significance was calculated using a linear mixed-model analysis with Bonferroni multiple comparisons. *P less than .05 vs control, $*P* less than .05 vs DHT.

**Figure 5.**
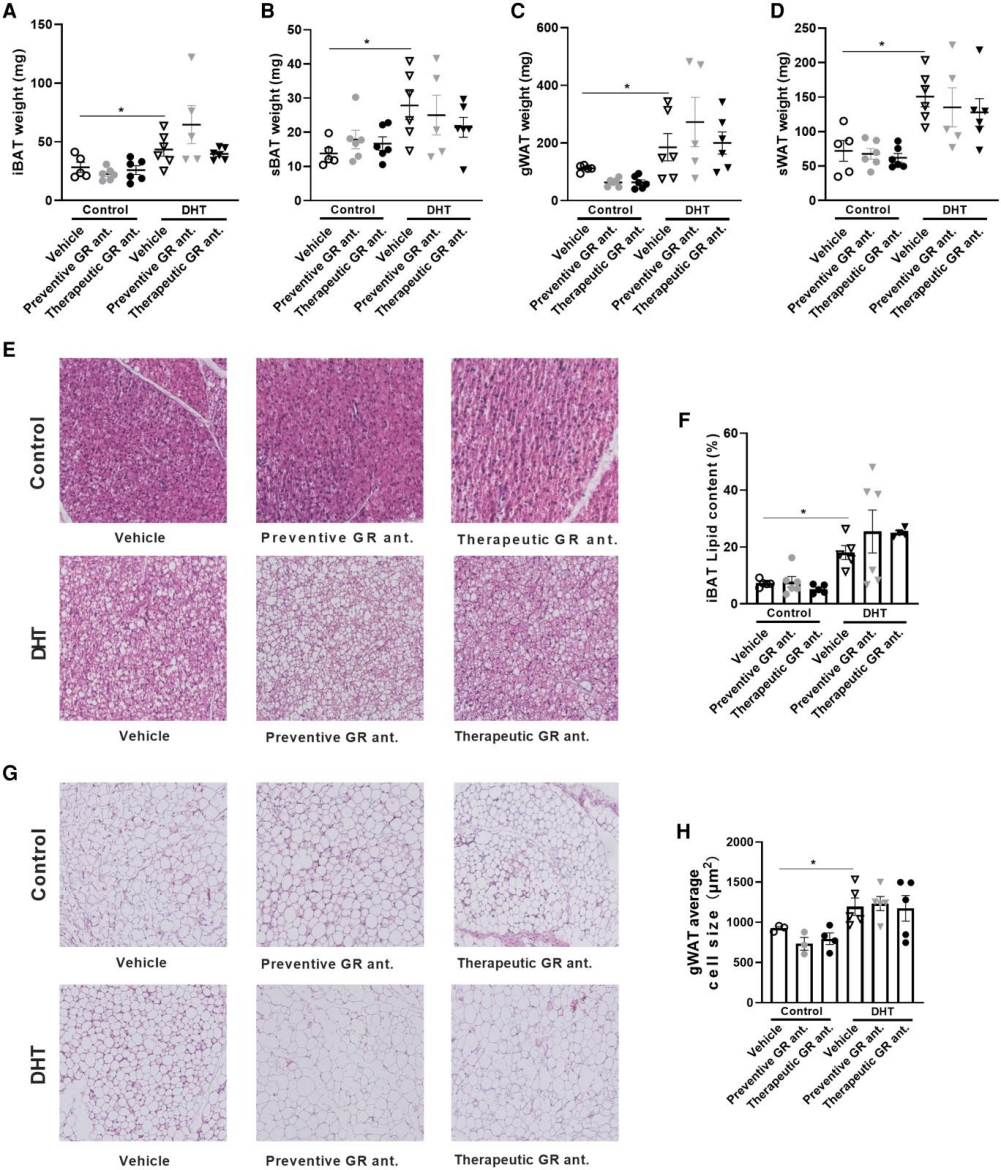
The effect of preventive and therapeutic glucocorticoid receptor (GR) antagonism on adipose tissue weight and lipid content of control and dihydrotestosterone (DHT)-exposed mice. The effect of preventive or therapeutic GR antagonism in control mice and DHT-exposed mice on A, iBAT weight; B, sBAT weight; C, gWAT weight; and D, sWAT weight. E, Representative histological images of hematoxylin and eosin–stained iBAT. F, iBAT lipid content. G, Representative histological images of hematoxylin and eosin–stained gWAT. H, Average adipocyte cell size. A to D, N = 5/6 per group; E and F, N = 4/5/6 per group; G and H, N = 3/4/5 per group. Statistical significance is calculated using 2-way analysis of variance followed by least significant difference post hoc test. *P less than .05 vs control.

We next investigated body weight and composition in control and DHT mice on preventive or therapeutic treatment with a GR antagonist. We observed that DHT exposure increased body weight as compared to control mice, and that preventive GR antagonism decreased total body weight in control and DHT-treated mice (Fig. 4A). On initiation of therapeutic treatment with the GR antagonist, we observed a reduction of body weight both in the control mice and the DHT-treated mice, although the effect appeared stronger in control than DHT-treated mice (Fig. 4B). When evaluating lean mass, we observed a significant increase in DHT-treated mice compared to control mice (Fig. 4C). Preventive treatment with the GR antagonist resulted in a decrease in lean mass both in control and DHT-treated mice (see Fig. 4C), while therapeutic GR antagonism reduced lean mass in control mice only (Fig. 4D). Both preventive and therapeutic treatment resulted in significant reduction in fat mass in control mice (Fig. 4E and 4F). DHT exposure increased fat mass, which was not further affected by preventive or therapeutic GR antagonism (see Fig. 4E and 4F). When evaluating the wet weight of different metabolic tissues, we found that DHT increased the weight of iBAT, sBAT, gWAT, and sWAT as compared to control mice, but that neither preventive nor therapeutic GR antagonism further influenced this (significant main effect of DHT but not of treatment; no statistical interaction; Fig. 5A-5D). In line with these findings, histological analysis of iBAT and gWAT showed increased iBAT lipid content and average adipocyte cell size in gWAT on DHT treatment, but no further effect by GR antagonism (significant main effects of DHT but not of treatment; no statistical interaction; Fig. 5E-5H).

### Preventive Glucocorticoid Receptor Antagonism Alleviates Hyperglycemia and Improves Glucose Tolerance in Dihydrotestosterone-exposed Mice

**Figure 6.**
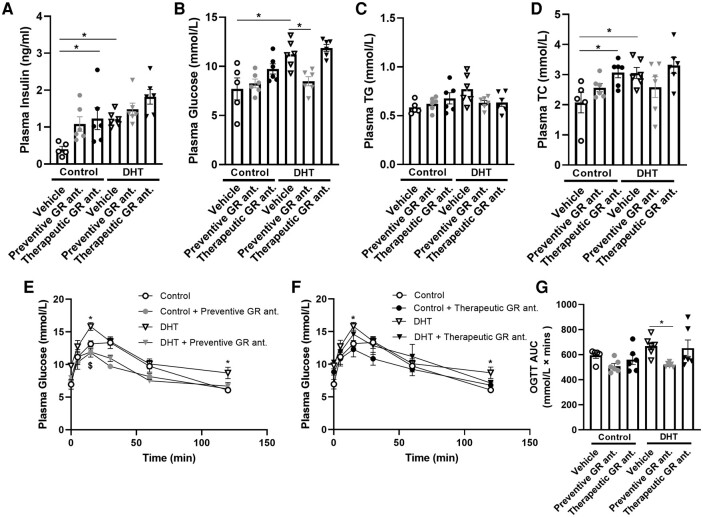
The effect of preventive and therapeutic glucocorticoid receptor (GR) antagonism on biochemistry and glucose tolerance of control and dihydrotestosterone (DHT)-exposed mice. Plasma levels after a 6-hour fast of A, insulin; B, glucose; C, triglycerides (TG); and D, total cholesterol (TC). E and F, Plasma glucose levels during an oral glucose tolerance test (OGTT) performed after a 6-hour fast in week 11. G, Area under the curve of glucose during OGTT. N = 5/6 per group. Statistical significance is calculated using 2-way analysis of variance followed by least significant difference post hoc test. *P less than .05 vs control, $*P* less than .05 vs DHT.

Analysis of plasma biochemistry showed that DHT exposure caused an increase in plasma insulin, glucose, and total cholesterol (main effects of DHT: *P* < .0006, *P* < .003, and *P* = .051, respectively), but no significant effect on triglyceride levels (Fig. 6A-6D). In control mice, we found that therapeutic GR antagonism significantly increased plasma insulin (see Fig. 6A). We observed that preventive treatment alleviated the DHT-induced increase in glucose (interaction effect *P* = .03; see Fig. 6B). Therapeutic GR antagonism increased plasma total cholesterol levels in control mice, with no clear effects in DHT-treated mice (see Fig. 6D). We performed an oral glucose tolerance test (OGTT) experiment in week 11 of the study to investigate glucose tolerance. DHT-treated mice exhibited a significant increase in plasma glucose levels as compared to control mice, both at 15 minutes and 120 minutes post glucose-bolus and on total glucose exposure (area under the curve) (Fig. 6E-6G). In DHT-treated mice, preventive GR antagonism resulted in a reduction in plasma glucose levels at 15 minutes (see Fig. 6E), while therapeutic treatment did not decrease plasma glucose level in control mice nor DHT-treated mice (see Fig. 6F). Preventive treatment with the GR antagonist readily lowered total glucose exposure in DHT-exposed mice (see Fig. 6G).

### Glucocorticoid Receptor Antagonism Increased Triglyceride-derived Fatty Acid Uptake in Adipose Tissues of Control Mice, Which Was Blunted in Dihydrotestosterone-exposed Mice

**Figure 7.**
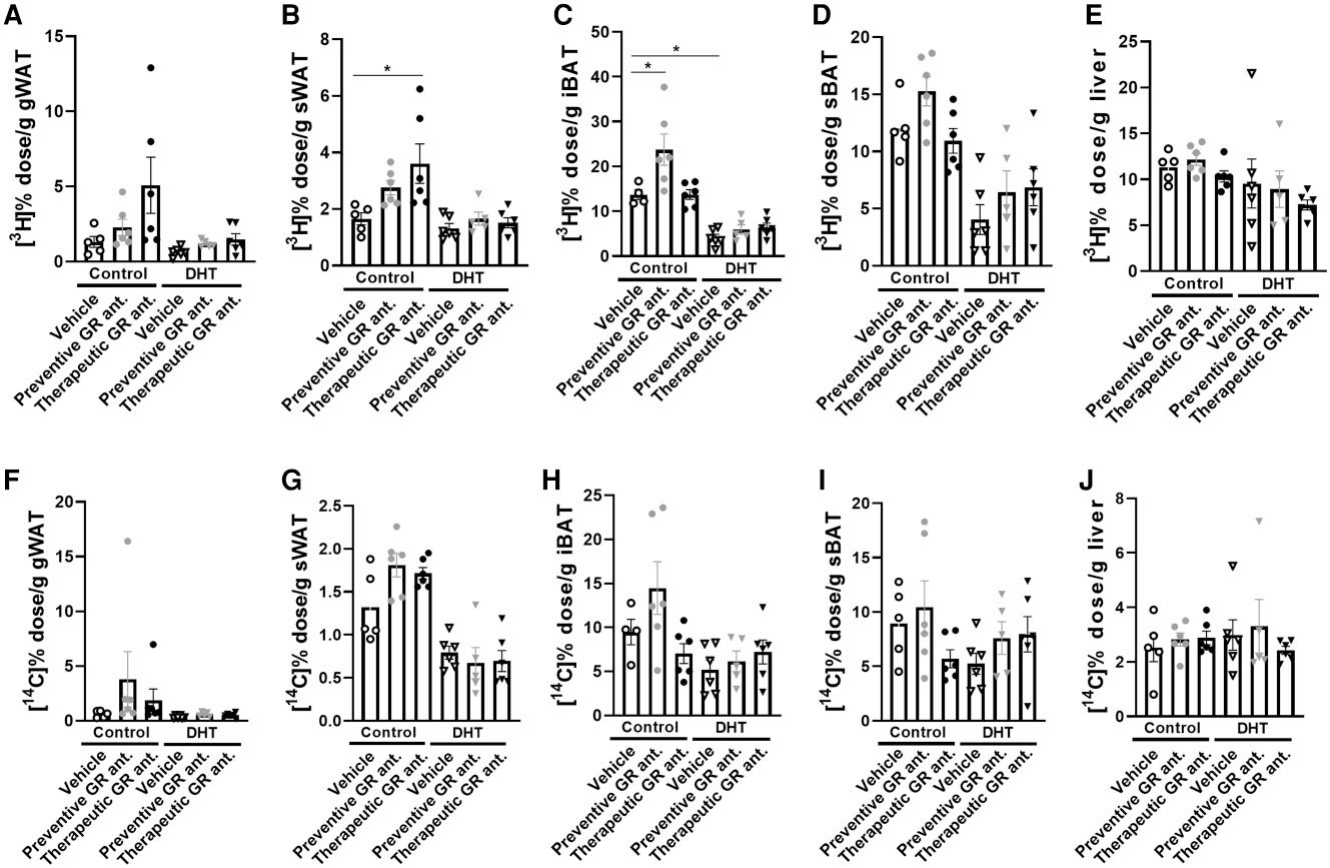
The effect of preventive and therapeutic glucocorticoid receptor (GR) antagonism on uptake of triglyceride-derived [^3^H]-labeled fatty acids and [^14^C]-labeled deoxyglucose in control and DHT-exposed mice. [^3^H] activity in A, gWAT; B, sWAT; C, iBAT; D, sBAT; and E, liver. [^14^C] activity in F, gWAT; G, sWAT; H, iBAT; I, sBAT; and J, liver. N = 5/6 per group. Statistical significance is calculated using 2-way way analysis of variance followed by least significant difference post hoc test. *P less than .05 vs control.

Both DHT and GR antagonist treatment affected triglyceride-derived fatty acid uptake by different tissues (Supplementary Table S2) [39]. We found in control mice that therapeutic GR antagonism seemed to increase [^3^H] activity in gWAT and significantly increased uptake in sWAT (Fig. 7A and 7B). Preventive treatment increased [^3^H] activity in iBAT and nonsignificantly in sBAT (Fig. 7C and 7D). DHT exposure in itself reduced [^3^H] activity in gWAT, sWAT, iBAT, sBAT, and liver as compared to control (significant main effects of DHT for all tissues; see Fig. 7A-7E). In DHT-treated mice, we observed that the effect of both preventive and therapeutic treatment with the GR antagonist on triglyceride-derived fatty acid uptake was completely blunted (see Fig. 7A-7C). When evaluating the uptake of [^14^C]-labeled deoxyglucose, DHT exposure reduced the uptake in sWAT and iBAT (significant main effects of DHT; Fig. 7G and 7H). We did not observe any other major effects on [^14^C] activity of GR antagonism (Fig. 7F-7J).

## Discussion

Women with PCOS have a higher risk of developing obesity and other metabolic disorders, and women with obesity show increased prevalence of PCOS [40, 41]. Many of the complications in PCOS are believed to be driven by hyperandrogenism, and previous studies show that a global loss of AR signaling fully protects female mice from the development of PCOS-like metabolic traits on excess androgen exposure [42]. However, many of the clinical features in PCOS are also characteristics of metabolic disorders driven by deregulated glucocorticoid signaling (eg, in Cushing syndrome) [43-45]. In addition to this, we previously observed (functional) crosstalk between glucocorticoid and androgen signaling [11] and we therefore investigated a possible role of GR signaling in (DHT-induced) metabolic features of PCOS capitalizing on the availability of the selective GR antagonist CORT125134 [33]. We confirmed the PCOS-associated features in our model of DHT exposure, including altered ovarian morphology with an increased number of unhealthy antral follicles and a decrease in granulosa cell layer thickness [46, 47]. GR antagonism did not influence any of these DHT-induced effects in the ovary, although our histological analysis of the ovary was underpowered, prohibiting any formal conclusion. It is important to note that while our model recapitulated many features of PCOS/prolonged DHT exposure, DHT-exposed mice did not develop liver steatosis (Supplementary Fig. S1 [39]), in contrast to previously reported data using a similar model of DHT exposure that was accompanied by overt liver steatosis [48]. Of note, our observation was based on biochemical measurements of triglycerides and total cholesterol, while histological determination of steatosis was not performed.

We observed that, after prolonged DHT exposure, the expression of many factors related to GR signaling were changed at the mRNA level. This includes hepatic expression of *Nr3c1* (coding for GR), *Hsd11b1*, and *Ncoa2*, which were all upregulated in DHT-treated mice. This shows that different aspects of glucocorticoid signaling, from receptor and coregulator expression to prereceptor metabolism of the ligand, are regulated by androgens. This is consistent with the previous findings that DHT treatment enhanced local concentrations of corticosterone in the liver [49]. Of note, we did not measure (hepatic) corticosterone levels directly in this study. In other tissues, we found little evidence of altered GR signaling in DHT-exposed mice, with the exception of gWAT and possibly sWAT, in which expression of *Hsd11b1* was increased, potentially resulting in increased local glucocorticoid (re)activation. The regulation of glucocorticoid-related factors like *Hsd11b1* by androgens could also play a role in metabolic physiology in women with PCOS, and likely results in elevated glucocorticoid turnover in tissues like WAT and liver. This could in turn (partially) contribute to the metabolic features observed in PCOS, including deregulated glucose metabolism. It is important to note that our mouse model of PCOS results in supraphysiological exposure of DHT, and the regulation of *Hsd11b1* expression and activity under more physiological androgen exposure requires further investigation. It was previously found that *HSD11B1* expression was elevated in ovaries of women with PCOS, as compared to non-PCOS patients [50]. This, in addition to the increased in hepatic and adipose expression of *Hsd11b1* observed in our mouse study, could contribute to elevated cortisol/corticosterone exposure in patients with PCOS. Indeed, patients with PCOS showed increased hair cortisol concentrations as compared to healthy women [51], possibly mediated via androgen regulation of *HSD11B1* expression.

Given the deregulated glucocorticoid signaling in the liver, we evaluated whether GR antagonism can prevent or alleviate DHT-induced metabolic features. For this we used a GR-specific antagonist, either administered continuously via diet-supplementation for a total period of 12 weeks (during the whole period of DHT treatment), or administered daily via oral gavage in mice with established DHT-induced metabolic symptoms. We found that preventive treatment with a GR antagonist alleviated the glucose levels during an OGTT, with significantly lower glucose exposure as compared to vehicle-treated DHT-exposed animals. On the other hand, we did not observe such improvement on therapeutic treatment with the GR antagonist. The differential effects of preventive and therapeutic GR antagonism may suggest that GR signaling is involved in the development of metabolic symptoms on DHT exposure, but that this does not necessarily provide a suitable therapeutic target when symptoms are fully established. We cannot exclude that a longer treatment duration with a GR antagonist may provide metabolic benefit. Importantly, the mode of administration of the GR antagonist was different between the preventive and therapeutic treatment groups, disallowing direct comparison as this may have resulted in differences in bioavailability and kinetics between the two treatment groups. We were unable to directly determine drug concentrations in plasma or target tissues, and it is therefore uncertain whether we approximated steady-state levels in the animals that received the compound via oral gavage, and if so, at what level.

For many other metabolic features that were observed in DHT-treated animals, neither preventive nor therapeutic GR antagonism showed improvement. We observed that DHT exposure induced lipid accumulation in adipose tissues, in line with previous findings in PCOS mouse models [47, 52, 53]. However, GR antagonism did not alter adipose tissue weight and lipid accumulation in DHT-treated mice. In other models for metabolic disease, benefits on metabolic health were observed on treatment with GR antagonists [54-58]. It thus seems that many activities of GR antagonists are lost in PCOS, possibly due to elevated androgen exposure that potentially takes over (part of) glucocorticoid effects. We indeed found that the uptake of triglyceride-derived fatty acids in adipose tissues was readily induced on GR antagonism in control mice, but that this was completely blunted in DHT-treated mice. Previous transcriptome studies showed that the large majority of GR-responsive genes are also regulated by AR [59], suggesting that GR-responsive transcripts can also be AR responsive. Furthermore, enhanced 5α-reductase activity has been reported in women with PCOS, leading to glucocorticoid degradation [60-62], and this may also result in abolished responsiveness to GR antagonists.

In summary, we found that GR antagonism improved glucose metabolism, but not other metabolic features, in a mouse model of elevated androgen exposure. The effects of GR antagonism on tissue uptake of triglyceride-derived fatty acids were lost in DHT-treated mice, showing that responsiveness to a GR antagonist may strongly depend on disease stage and context.

## Disclosures

O.C.M. receives research funding from Corcept Therapeutics, a pharmaceutical company that commercially develops CORT125134. J.K. is seconded to Corcept Therapeutics for 50% of his time. The other authors have nothing to disclose.

## Data Availability

Some or all data sets generated during and/or analyzed during the current study are not publicly available but are available from the corresponding author on reasonable request.

## Abbreviations

11β-HSD1: 11 beta-hydroxysteroid dehydrogenase type 1
AR: androgen receptor
DHT: dihydrotestosterone
GR: glucocorticoid receptor
gWAT: gonadal white adipose tissue
iBAT: interscapular brown adipose tissue
mRNA: messenger RNA
OGTT: oral glucose tolerance test
PBS: phosphate-buffered saline
PCOS: polycystic ovary syndrome
PR: progesterone receptor
sBAT: subscapular brown adipose tissue
sWAT: subcutaneous white adipose tissue

## References

[1] Dumesic DA, Oberfield SE, Stener-Victorin E, Marshall JC, Laven JS, Legro RS. Scientific statement on the diagnostic criteria, epidemiology, pathophysiology, and molecular genetics of polycystic ovary syndrome. Endocr Rev. 2015;36(5):487‐525. 26426951 10.1210/er.2015-1018 PMC4591526

[2] Meier RK . Polycystic ovary syndrome. Nurs Clin North Am. 2018;53(3):407‐420. 30100006 10.1016/j.cnur.2018.04.008

[3] Azziz R, Carmina E, Dewailly D, et al The androgen excess and PCOS society criteria for the polycystic ovary syndrome: the complete task force report. Fertil Steril. 2009;91(2):456‐488. 18950759 10.1016/j.fertnstert.2008.06.035

[4] Goodarzi MO, Dumesic DA, Chazenbalk G, Azziz R. Polycystic ovary syndrome: etiology, pathogenesis and diagnosis. Nat Rev Endocrinol. 2011;7(4):219‐231. 21263450 10.1038/nrendo.2010.217

[5] Pappalardo MA, Russo GT, Pedone A, et al Very high frequency of the polymorphism for the insulin receptor substrate 1 (IRS-1) at codon 972 (glycine972arginine) in southern Italian women with polycystic ovary syndrome. Horm Metab Res. 2010;42 (08):575‐584. 20229450 10.1055/s-0030-1249020

[6] Pappalardo MA, Vita R, Di Bari F, Le Donne M, Trimarchi F, Benvenga S. Gly972Arg of IRS-1 and Lys121Gln of PC-1 polymorphisms act in opposite way in polycystic ovary syndrome. J Endocrinol Invest. 2017;40(4):367‐376. 27785750 10.1007/s40618-016-0569-7

[7] Caldwell ASL, Eid S, Kay CR, et al Haplosufficient genomic androgen receptor signaling is adequate to protect female mice from induction of polycystic ovary syndrome features by prenatal hyperandrogenization. Endocrinology. 2015;156(4):1441‐1452. 25643156 10.1210/en.2014-1887

[8] Xiong T, Rodriguez Paris V, Edwards MC, et al Androgen signaling in adipose tissue, but less likely skeletal muscle, mediates development of metabolic traits in a PCOS mouse model. Am J Physiol Endocrinol Metab. 2022;323(2):E145‐E158. 35658542 10.1152/ajpendo.00418.2021

[9] Lu NZ, Wardell SE, Burnstein KL, et al International union of pharmacology. LXV. The pharmacology and classification of the nuclear receptor superfamily: glucocorticoid, mineralocorticoid, progesterone, and androgen receptors. Pharmacol Rev. 2006;58(4):782‐797. 17132855 10.1124/pr.58.4.9

[10] Roy AK, Lavrovsky Y, Song CS, et al Regulation of androgen action. Vitam Horm. 1999;55:309‐352. 9949684 10.1016/s0083-6729(08)60938-3

[11] Spaanderman DCE, Nixon M, Buurstede JC, et al Androgens modulate glucocorticoid receptor activity in adipose tissue and liver. J Endocrinol. 2018;240:51‐63. 10.1530/JOE-18-0503 30400038

[12] Hayashi R, Okuno Y, Mukai K, et al Adipocyte GR inhibits healthy adipose expansion through multiple mechanisms in cushing syndrome. Endocrinology. 2019;160(3):504‐521. 30649271 10.1210/en.2018-01029

[13] Lu Y, Wang E, Chen Y, et al Obesity-induced excess of 17-hydroxyprogesterone promotes hyperglycemia through activation of glucocorticoid receptor. J Clin Invest. 2020;130(7):3791‐3804. 32510471 10.1172/JCI134485 PMC7324200

[14] Peckett AJ, Wright DC, Riddell MC. The effects of glucocorticoids on adipose tissue lipid metabolism. Metab Clin Exp. 2011;60(11):1500‐1510. 21864867 10.1016/j.metabol.2011.06.012

[15] Exton JH . Regulation of gluconeogenesis by glucocorticoids. Monogr Endocrinol. 1979;12:535‐546. 386091 10.1007/978-3-642-81265-1_28

[16] Kraus-Friedmann N . Hormonal regulation of hepatic gluconeogenesis. Physiol Rev. 1984;64(1):170‐259. 6141578 10.1152/physrev.1984.64.1.170

[17] Exton JH, Friedmann N, Wong EH, Brineaux JP, Corbin JD, Park CR. Interaction of glucocorticoids with glucagon and epinephrine in the control of gluconeogenesis and glycogenolysis in liver and of lipolysis in adipose tissue. J Biol Chem. 1972;247(11):3579‐3588. 4337859

[18] Stewart PM, Krozowski ZS. 11 beta-Hydroxysteroid dehydrogenase. Vitam Horm. 1999;57:249‐324. 10232052

[19] Walker BR, Andrew R. Tissue production of cortisol by 11beta-hydroxysteroid dehydrogenase type 1 and metabolic disease. Ann N Y Acad Sci. 2006;1083(1):165‐184. 17148739 10.1196/annals.1367.012

[20] Gathercole LL, Stewart PM. Targeting the pre-receptor metabolism of cortisol as a novel therapy in obesity and diabetes. J Steroid Biochem Mol Biol. 2010;122(1-3):21‐27. 20347978 10.1016/j.jsbmb.2010.03.060

[21] Zhang Y, Calvo E, Martel C, Luu-The V, Labrie F, Tchernof A. Response of the adipose tissue transcriptome to dihydrotestosterone in mice. Physiol Genomics. 2008;35(3):254‐261. 18728228 10.1152/physiolgenomics.00257.2007

[22] Zhu L, Hou M, Sun B, et al Testosterone stimulates adipose tissue 11beta-hydroxysteroid dehydrogenase type 1 expression in a depot-specific manner in children. J Clin Endocrinol Metab. 2010;95(7):3300‐3308. 20410225 10.1210/jc.2009-2708

[23] Mangelsdorf DJ, Thummel C, Beato M, et al The nuclear receptor superfamily: the second decade. Cell. 1995;83(6):835‐839. 8521507 10.1016/0092-8674(95)90199-x PMC6159888

[24] Xu J, Li Q. Review of the in vivo functions of the p160 steroid receptor coactivator family. Mol Endocrinol. 2003;17(9):1681‐1692. 12805412 10.1210/me.2003-0116

[25] Duteil D, Chambon C, Ali F, et al The transcriptional coregulators TIF2 and SRC-1 regulate energy homeostasis by modulating mitochondrial respiration in skeletal muscles. Cell Metab. 2010;12(5):496‐508. 21035760 10.1016/j.cmet.2010.09.016 PMC3032428

[26] Jeong JW, Kwak I, Lee KY, et al The genomic analysis of the impact of steroid receptor coactivators ablation on hepatic metabolism. Mol Endocrinol. 2006;20(5):1138‐1152. 16423883 10.1210/me.2005-0407

[27] Picard F, Géhin M, Annicotte J, et al SRC-1 and TIF2 control energy balance between white and brown adipose tissues. Cell. 2002;111(7):931‐941. 12507421 10.1016/s0092-8674(02)01169-8

[28] Coste A, Louet JF, Lagouge M, et al The genetic ablation of SRC-3 protects against obesity and improves insulin sensitivity by reducing the acetylation of PGC-1alpha. Proc Natl Acad Sci U S A. 2008;105(44):17187‐17192. 18957541 10.1073/pnas.0808207105 PMC2579399

[29] Broekema MF, Hollman DAA, Koppen A, et al Profiling of 3696 nuclear receptor-coregulator interactions: a resource for biological and clinical discovery. Endocrinology. 2018;159(6):2397‐2407. 29718163 10.1210/en.2018-00149

[30] Nikolić M, Macut D, Djordjevic A, et al Possible involvement of glucocorticoids in 5alpha-dihydrotestosterone-induced PCOS-like metabolic disturbances in the rat visceral adipose tissue. Mol Cell Endocrinol. 2015;399:22‐31. 25179821 10.1016/j.mce.2014.08.013

[31] Vitellius G, Trabado S, Bouligand J, Delemer B, Lombes M. Pathophysiology of glucocorticoid signaling. Ann Endocrinol (Paris). 2018; 79(3):98‐106. 29685454 10.1016/j.ando.2018.03.001

[32] Hunt H, Donaldson K, Strem M, et al Assessment of safety, tolerability, pharmacokinetics, and pharmacological effect of orally administered CORT125134: an adaptive, double-blind, randomized, placebo-controlled phase 1 clinical study. Clin Pharmacol Drug Dev. 2018;7(4):408‐421. 28967708 10.1002/cpdd.389 PMC5947602

[33] Viho EMG, Kroon J, Feelders RA, et al Peripheral glucocorticoid receptor antagonism by relacorilant with modest HPA axis disinhibition. J Endocrinol. 2023;256(2):e220263. 36445262 10.1530/JOE-22-0263 PMC9874980

[34] Caldwell AS, Middleton LJ, Jimenez M, et al Characterization of reproductive, metabolic, and endocrine features of polycystic ovary syndrome in female hyperandrogenic mouse models. Endocrinology. 2014;155(8):3146‐3159. 24877633 10.1210/en.2014-1196

[35] Aflatounian A, Edwards MC, Rodriguez Paris V, et al Androgen signaling pathways driving reproductive and metabolic phenotypes in a PCOS mouse model. J Endocrinol. 2020;245(3):381‐395. 32229702 10.1530/JOE-19-0530

[36] Rensen PC, van Dijk MC, Havenaar EC, Bijsterbosch MK, Kruijt JK, van Berkel TJ. Selective liver targeting of antivirals by recombinant chylomicrons–a new therapeutic approach to hepatitis B. Nat Med. 1995;1(3):221‐225. 7585037 10.1038/nm0395-221

[37] Khedoe PPSJ, Hoeke G, Kooijman S, et al Brown adipose tissue takes up plasma triglycerides mostly after lipolysis. J Lipid Res. 2015;56(1):51‐59. 25351615 10.1194/jlr.M052746 PMC4274071

[38] Kooijman S, Boon MR, Parlevliet ET, et al Inhibition of the central melanocortin system decreases brown adipose tissue activity. J Lipid Res. 2014;55(10):2022‐2032. 25016380 10.1194/jlr.M045989 PMC4173995

[39] Kroon J . Li Journal of the Endocrine Society Supplementary Materials. figshare. 2023. Doi: 10.6084/m9.figshare.24032331.v3

[40] Lim SS, Davies MJ, Norman RJ, Moran LJ. Overweight, obesity and central obesity in women with polycystic ovary syndrome: a systematic review and meta-analysis. Hum Reprod Update. 2012;18(6):618‐637. 22767467 10.1093/humupd/dms030

[41] Moran LJ, Norman RJ, Teede HJ. Metabolic risk in PCOS: phenotype and adiposity impact. Trends Endocrinol Metab. 2015;26(3):136‐143. 25591984 10.1016/j.tem.2014.12.003

[42] Caldwell ASL, Edwards MC, Desai R, et al Neuroendocrine androgen action is a key extraovarian mediator in the development of polycystic ovary syndrome. Proc Natl Acad Sci U S A. 2017;114(16):E3334‐E3343. 28320971 10.1073/pnas.1616467114 PMC5402450

[43] Lacroix A, Feelders RA, Stratakis CA, Nieman LK. Cushing's syndrome. Lancet. 2015;386(9996):913‐927. 26004339 10.1016/S0140-6736(14)61375-1

[44] Beaupere C, Liboz A, Fève B, Blondeau B, Guillemain G. Molecular mechanisms of glucocorticoid-induced insulin resistance. Int J Mol Sci. 2021;22(2):623. 33435513 10.3390/ijms22020623 PMC7827500

[45] Giordano C, Guarnotta V, Pivonello R, et al Is diabetes in cushing's syndrome only a consequence of hypercortisolism? Eur J Endocrinol. 2014;170(2):311‐319. 24255133 10.1530/EJE-13-0754

[46] Walters KA, Rodriguez Paris V, Aflatounian A, Handelsman DJ. Androgens and ovarian function: translation from basic discovery research to clinical impact. J Endocrinol. 2019;242(2):R23‐R50. 31125975 10.1530/JOE-19-0096

[47] van Houten ELAF, Kramer P, McLuskey A, Karels B, Themmen APN, Visser JA. Reproductive and metabolic phenotype of a mouse model of PCOS. Endocrinology. 2012;153(6):2861‐2869. 22334715 10.1210/en.2011-1754

[48] Cox MJ, Edwards MC, Rodriguez Paris V, et al Androgen action in adipose tissue and the brain are key mediators in the development of PCOS traits in a mouse model. Endocrinology. 2020;161(7):baqq061. 10.1210/endocr/bqaa061 32301482

[49] Vojnović Milutinović D, Teofilović A, Velićković N, et al Glucocorticoid signaling and lipid metabolism disturbances in the liver of rats treated with 5alpha-dihydrotestosterone in an animal model of polycystic ovary syndrome. Endocrine. 2021;72(2):562‐572. 33449293 10.1007/s12020-020-02600-1

[50] Li X, Hu S, Zhu Q, et al Addressing the role of 11beta-hydroxysteroid dehydrogenase type 1 in the development of polycystic ovary syndrome and the putative therapeutic effects of its selective inhibition in a preclinical model. Metab Clin Exp. 2021;119:154749. 33722534 10.1016/j.metabol.2021.154749

[51] Gonzalez D, Maidana P, Ibar C, et al Hair cortisol in polycystic ovary syndrome. Sci Rep. 2022;12(1):10309. 35725989 10.1038/s41598-022-14061-9 PMC9209522

[52] Rodriguez Paris V, Edwards MC, Aflatounian A, et al Pathogenesis of reproductive and metabolic PCOS traits in a mouse model. J Endocr Soc. 2021;5(6):bvab060. 34056500 10.1210/jendso/bvab060 PMC8152184

[53] Torres PJ, Ho BS, Arroyo P, et al Exposure to a healthy gut microbiome protects against reproductive and metabolic dysregulation in a PCOS mouse model. Endocrinology. 2019;160(5):1193‐1204. 30924862 10.1210/en.2019-00050 PMC6482036

[54] Kroon J, Viho EMG, Gentenaar M, et al The development of novel glucocorticoid receptor antagonists: from rational chemical design to therapeutic efficacy in metabolic disease models. Pharmacol Res. 2021;168:105588. 33798733 10.1016/j.phrs.2021.105588

[55] Asagami T, Belanoff JK, Azuma J, Blasey CM, Clark RD, Tsao PS. Selective glucocorticoid receptor (GR-II) antagonist reduces body weight gain in mice. J Nutr Metab. 2011;2011:235389. 21811679 10.1155/2011/235389 PMC3146995

[56] Jacobson PB, von Geldern TW, Ohman L, et al Hepatic glucocorticoid receptor antagonism is sufficient to reduce elevated hepatic glucose output and improve glucose control in animal models of type 2 diabetes. J Pharmacol Exp Ther. 2005;314(1):191‐200. 15784656 10.1124/jpet.104.081257

[57] Pivonello R, Bancos I, Feelders RA, et al Relacorilant, a selective glucocorticoid receptor modulator, induces clinical improvements in patients with cushing syndrome: results from A prospective, open-label phase 2 study. Front Endocrinol (Lausanne). 2021;12:662865. 34335465 10.3389/fendo.2021.662865 PMC8317576

[58] Koorneef LL, Kroon J, Viho EMG, et al The selective glucocorticoid receptor antagonist CORT125281 has tissue-specific activity. J Endocrinol. 2020;246(1):79‐92. 32369774 10.1530/JOE-19-0486 PMC7274539

[59] Arora VK, Schenkein E, Murali R, et al Glucocorticoid receptor confers resistance to antiandrogens by bypassing androgen receptor blockade. Cell. 2013;155(6):1309‐1322. 24315100 10.1016/j.cell.2013.11.012 PMC3932525

[60] Chin D, Shackleton C, Prasad VK, et al Increased 5alpha-reductase and normal 11beta-hydroxysteroid dehydrogenase metabolism of C19 and C21 steroids in a young population with polycystic ovarian syndrome. J Pediatr Endocrinol Metab. 2000;13(3):253‐259. 10714750 10.1515/jpem.2000.13.3.253

[61] Fassnacht M, Schlenz N, Schneider SB, Wudy SA, Allolio B, Arlt W. Beyond adrenal and ovarian androgen generation: increased peripheral 5 alpha-reductase activity in women with polycystic ovary syndrome. J Clin Endocrinol Metab. 2003;88(6):2760‐2766. 12788885 10.1210/jc.2002-021875

[62] Blumenfeld Z, Kaidar G, Zuckerman-Levin N, Dumin E, Knopf C, Hochberg Z. Cortisol-Metabolizing enzymes in polycystic ovary syndrome. Clin Med Insights Reprod Health. 2016;10:9‐13. 27168731 10.4137/CMRH.S35567 PMC4859446

